# Molecular Mechanisms and Biochemical Pathways for Micronutrient Acquisition and Storage in Legumes to Support Biofortification for Nutritional Security

**DOI:** 10.3389/fpls.2021.682842

**Published:** 2021-06-07

**Authors:** Manish Roorkiwal, Sarita Pandey, Dil Thavarajah, R. Hemalatha, Rajeev K. Varshney

**Affiliations:** ^1^International Crops Research Institute for the Semi-Arid Tropics (ICRISAT), Hyderabad, India; ^2^The UWA Institute of Agriculture, The University of Western Australia, Perth, WA, Australia; ^3^Plant and Environmental Sciences, Poole Agricultural Center, Clemson University, Clemson, SC, United States; ^4^ICMR-National Institute of Nutrition (NIN), Hyderabad, India; ^5^State Agricultural Biotechnology Centre, Centre for Crop and Food Innovation, Murdoch University, Murdoch, WA, Australia

**Keywords:** malnutrition, micronutrient, metabolic pathway, biofortification, trait-mapping, genomics

## Abstract

The world faces a grave situation of nutrient deficiency as a consequence of increased uptake of calorie-rich food that threaten nutritional security. More than half the world’s population is affected by different forms of malnutrition. Unhealthy diets associated with poor nutrition carry a significant risk of developing non-communicable diseases, leading to a high mortality rate. Although considerable efforts have been made in agriculture to increase nutrient content in cereals, the successes are insufficient. The number of people affected by different forms of malnutrition has not decreased much in the recent past. While legumes are an integral part of the food system and widely grown in sub-Saharan Africa and South Asia, only limited efforts have been made to increase their nutrient content in these regions. Genetic variation for a majority of nutritional traits that ensure nutritional security in adverse conditions exists in the germplasm pool of legume crops. This diversity can be utilized by selective breeding for increased nutrients in seeds. The targeted identification of precise factors related to nutritional traits and their utilization in a breeding program can help mitigate malnutrition. The principal objective of this review is to present the molecular mechanisms of nutrient acquisition, transport and metabolism to support a biofortification strategy in legume crops to contribute to addressing malnutrition.

## Introduction

Combating malnutrition in all its forms is one of the most significant global health challenges of the 21st century as it affects mostly women, infants, children, and adolescents. The World Health Organization (WHO) estimates that globally, more than 2 billion people suffer from micronutrient malnutrition, also known as “hidden hunger” (Ritchie and Roser, 2020). Modern breeding approaches, together with best agronomic practices during the Green Revolution, were instrumental in significantly increasing the production of major cereal crops. This increase led to a reduction in global hunger. However, several developing countries still face challenges related to malnutrition due to the consumption of cereal-based diets. Poor diets devoid of nutrient concentrations and bioavailability are among the principal risk factors for non-communicable diseases (NCDs), responsible for about 70% of deaths in 2015 (Forouzanfar et al., 2015).

### Malnutrition Across the World

Malnutrition refers to inadequacies, excesses, or imbalances in an individual’s consumption of nutrients that adversely affect health and ultimately impair growth and fitness. It can be classified into three broad forms: undernutrition (wasting, stunting, and underweight), micronutrient-linked malnutrition (lack or excess of vitamins or and minerals) and overweight (obesity). It affects humans through increased morbidity, disability, stunted mental growth, and reduced National Socio-economic Development Plan (NSEDP) (FAO et al., 2017). Disturbingly, every second pregnant woman and about 40% of pre-school children in developing countries are estimated to be anemic; this leads to 20% of all maternal deaths. WHO estimated 5.3 million child deaths under the age of 5 during 2018, of which around 45% were linked to undernutrition.

Similarly, the share of neonatal deaths is projected to increase from roughly 46% in 2016 to 52% in 2030 (WHO, 2017). To make matters worse, around 2 billion people worldwide are anemic, mainly due to iron (Fe) deficiency (WHO, 2017). About 32.8% of women of reproductive stage and 32.5% of non-pregnant women, and 41.7% of children under the age of 5 are suffering from anemia globally (World Bank Data, 2016). Additionally, apart from zinc, iodine, and vitamin A deficiency, calcium, magnesium, and copper deficiencies are also prevalent in many developed and developing countries (Kumssa et al., 2015). Malnutrition has multifaceted consequences as it increases medical expenses and reduces productivity and economic growth. Malnutrition accounts for 11% of GDP losses in Asia and Africa, which is higher than the GDP loss experienced during the 2008–2010 financial crisis (Von Grebmer et al., 2016). Focusing only on delivering the energy needs of resource-poor people without considering their nutrient requirements will exacerbate the current state of malnutrition (Zarocostas, 2009). The Green Revolution was successful in increasing the productivity of major cereal crops multifold, preventing widespread famines and increasing the profitability of farmers in many developing countries (Bouis and Welch, 2010). However, a rise in micronutrient malnutrition in many nations suggests that agriculture needs to reevaluate its strategy to provide a healthy mix of sufficient calories along with essential nutrients. A sustainable solution to malnutrition would lie in linking agriculture to nutrition and health (Jones and Ejeta, 2016).

### Human Nutrient Requirements

A human body needs more than 50 macronutrients (>0.1 % of dry mass) and micronutrients (<0.01 % of dry weight) from five groups of essential nutrients for proper growth and development (Table 1). Though these nutrients are needed in meager quantities, they enable the body to produce enzymes, hormones, and other essential substances that aid growth and development. The recommended dietary allowance (RDA) for various nutrients varies with gender and age (Supplementary File 1). Along with oxygen, water and carbohydrates, vitamins and minerals are vital substances for our bodies to develop and function properly. According to the National Center for Complementary and Integrative Health (NCCIH, 2018) thirteen known vitamins, namely A, C, D, E, and K, and B vitamins (B1, B2, B3, B5, B7, B6, B12, and B9) and fifteen minerals, namely Calcium (Ca), Phosphorus (P), Potassium (K), Sodium (Na), Chlorine (Cl), Magnesium (Mg), Iron (Fe), Zinc (Zn), Iodine (I), Chromium (Cr), Copper (Cu), Fluorine (F), Molybdenum (Mo), Manganese (Mn), and Sulfur (S) are essential for health. Researchers have highlighted the need for 22 minerals for human well-being (White and Broadley, 2009), the lack of which present a grave threat to the health and development of populations around the globe, especially children and pregnant women in low-income countries.

**TABLE 1:** Five groups of essential nutrients for human life.

Basic	Amino Acids	Lipids-Fat	Minerals	Vitamins
Oxygen, water, Carbohydrates	Histidine, Isolueucine, Leucine, Lysine, Methionine, Phenylalanine, Threonine, Tryptophan, Valine	Linoleic acid, Linolenic acid	Na, K, Ca, Mg, *S*, P, Cl, Cr, Fe, Zn, Cu, Mn, I, F, Se, Mo, Co, *B*, *Ni*, *Si*, *As*, *Sn*	A, D, E, K, C, B_1_, B_2_, B_3_, B_5_, B_6_, B_7_, B_9_, B_12_

### Role of Legumes in Eradicating Malnutrition

**FIGURE 1:**
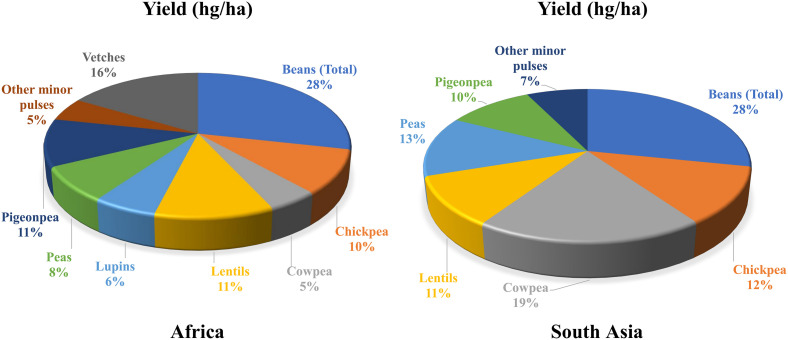
Share (%) of legumes’ yields in South Asia and Africa. Source: http://www.fao.org/faostat/en/#data/QC; accessed on April 16, 2018 Data Year: 2016. South Asia: Afghanistan, Bangladesh, Bhutan, India, Maldives, Nepal, Pakistan, and Sri Lanka. Africa: Algeria, Benin, Burkina Faso, Burundi, Cabo Verde, Cameroon, Central African Republic, Chad, Comoros, Congo, Côte d’Ivoire, Democratic Republic of the Congo, Djibouti, Egypt, Eritrea, Ethiopia, Gabon, Gambia, Guinea, Guinea-Bissau, Kenya, Lesotho, Liberia, Libya, Madagascar, Malawi, Mali, Mauritania, Morocco, Mozambique, Namibia, Niger, Nigeria, Réunion, Rwanda, Senegal, Sierra Leone, Somalia, South Africa, Togo, Sudan, Swaziland, Tunisia, Uganda, United Republic of Tanzania, Zambia, and Zimbabwe.

Malnutrition and poverty are closely related, which is also evident from FAO’s data on per capita income and level of malnutrition^1^. Around 82% of the extremely poor live in South Asia (SA) and sub-Saharan Africa (SSA), regions that host countries severely affected by one or other form of malnutrition. Considering the substantial socio-economic impact of legumes in these regions, their importance for food and nutritional security has been realized (Figure 1). In general, legume seeds have higher concentrations of essential minerals, vitamins, and protein than those of cereals (White and Broadley, 2009).

Legumes are an inexpensive source of protein (20–25%), minerals (Fe, Mg, K, P, and Zn) and vitamins (B1, B2, B3, B6, and B9) available to hundreds of millions of resource-poor people in SA and SSA. They are exceptionally notable because they complement the starches derived from cereals and root crops and help in efficient nutrient absorption. Legumes have a low-glycemic index and are rich in dietary fiber (8–27.5%), of which 3.3–13.8% correspond to soluble fiber (Sánchez-Chino et al., 2015). Storage protein is the major fraction of proteins in legumes. Almost 70% of the total protein comprises globulin, 10–20% each albumin and glutelins and low levels of prolamins (Sharif et al., 2018). Based on their lipid content, legumes can be classified into two main groups: those with low-fat content (1–6%), such as chickpea, lentil, bean, broad bean, etc., (Sánchez-Chino et al., 2015), and those with a high concentration of fat, which includes peanut and soybean (50 and 18%, respectively). The nutritional properties of legumes and their importance have been extensively reviewed in many articles (Jukanti et al., 2012; Mudryj et al., 2014; Sánchez-Chino et al., 2015; Foyer et al., 2016).

Considering the challenges related to hidden hunger and non-availability of nutritious food to a major portion of the global population, it is high time to initiate crop biofortification efforts. More than half of the world’s population faces impaired growth and fitness due to imbalances in an individual’s consumption of nutrients. Micronutrient malnutrition exists in several Asian and SSA countries due to the consumption of mainly cereals-based diets deprived of nutrient concentrations and bioavailability. Agriculture efforts focus on increasing nutrient content in cereals, but these are not enough to meet the global nutrition standards (Finkelstein et al., 2017). Legume crops have good genetic variation for nutritional traits, and therefore, there is a high potential for enhancing nutrients in seeds to contribute toward nutritional security. In this context, the discovery of genes and pathways accountable for nutrient acquisition and transport is pre-requisite. The targeted identification of precise factors related to nutritional traits and their utilization in a breeding program can help mitigate the challenge of malnutrition. A biofortification strategy by deploying breeding approaches for the nutritional improvement and microbiome for agronomic trait improvement in legumes has been presented (Rehman et al., 2019). However, it does not discuss molecular mechanisms and biochemical pathways for micronutrient acquisition and storage in legumes. This MS, therefore, provides up-to-date information on nutrient uptake mechanism and metabolism process that will serve as a foundation to initiate legume biofortification programs by integrating modern breeding approaches.

## Molecular Mechanisms of Mineral Acquisition and Transport

In order to maximize the bioavailability of nutrients, it is essential to understand the process of mineral acquisition, transport, and accumulation in legume seeds. Each of these processes is probably controlled by some genes, many of which are yet to be identified. Several studies have identified genes involved in translocation to different vegetative tissues and ultimately to seeds (Sperotto et al., 2014; Jeong et al., 2017). However, there is very limited knowledge of phloem-expressed genes involved in mineral loading and mobilization to different sink tissues (Braun et al., 2014). Therefore, while studies on specific transporters help us understand their function, whole-plant studies are required to ascertain transporters most relevant to seed mineral delivery. The acquisition and the mobilization of minerals in plants have been broadly studied (Walker and Waters, 2011; González-Guerrero et al., 2016; Xue et al., 2016). Several stresses can lead to the non-availability of key nutrition factors and result in improper crop growth. Details about these different stresses and their effect and a potential solution are provided in Table 2.

**TABLE 2:** Constraints to nutrient uptake, transport, storage, and effective survival strategies.

Stress condition	Constraint	Potential solution	References
High pH, salinity, and carbonate content of soil	Iron non-availability leading to Iron deficiency chlorosis (IDC) Limited ion movement to transpiration stream Reduced shoot growth	Coordinate expression of an active proton pump to increase solubility of Fe^+3^, a ferric chelate reductase to generate the more soluble Fe^+2^, and finally an iron transporter	Hell and Stephan, 2003
Mineral toxicity	Cytotoxicity Limited uptake	Compartmentalization of minerals Outflow of organic ions for chelation of toxic ions	Socha and Guerinot, 2014
Mn toxicity	Mn toxicity can arise in acidic and poorly drained soil Mn can compete and prevent uptake of other essential elements (Ca, Mg, Fe, and P)	Sequestering of Mn in the apoplast or vacuole	Millaleo et al., 2013
Mineral deficiency in soil	Inadequate nutrient acquisition	Enhanced uptake by transporters and developmental adaptation Root architecture re-modeling for efficient acquisition of minerals Partitioning for storage of minerals	Mickelbart et al., 2015
Nutrient retention and bioavailability	Inadequate nutrient in seeds	Improved post-harvest processing and cooking methods and conditions and duration of storage Screening of promising lines for micronutrient bioavailability Detect and understand plant biosynthetic genes and pathways of nutritional importance, including those for nutrient absorption enhancers and inhibitors	Nestel et al., 2006
Anti-nutrients (Phytic acid, Trypsin inhibitors, etc.)	Low bioavailability	Soaking of legumes before cooking Food diversification Development of genotypes with low anti-nutrients	Xie et al., 2017
Lack or deficiency of promoters like inulin, histidine, lysine, etc.	Low bioavailability of nutrients	Selection of genotypes with high level of promoters Development of genotypes with high promoters, like inulin, etc.	White and Broadley, 2005

### Iron (Fe) Transport

**FIGURE 2:**
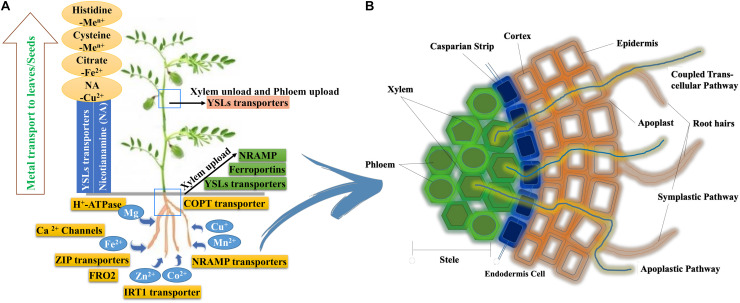
**(A)** The different transporters in the uptake of nutrients from the soil and their translocation to aerial parts. **(B)** A schematic representation of mineral transport to roots through different pathways.

Legumes are “strategy I” plants that acidify the rhizosphere through an H^+^-ATPase (the enzyme of HA2, H^+^-ATPase family) to increase Fe^3+^ solubility (Santi and Schmidt, 2009). Then they reduce Fe^3+^ to Fe^2+^ with the help of chelate reductase, ferric reduction oxidase (*FRO2*)and finally Fe^2+^ taken up by root’s plasma membrane through a Fe^2+^ iron-regulated transporter (*IRT1*) or its homologues such as natural resistance-associated macrophage protein 1 (*NRAMP1*) or divalent metal-ion transporter 1 (*DMT1*) (Figure 2). Rhizosphere acidification is mainly associated with the release of protons followed by surplus uptake of cations (Fe^+^) over anions during nitrogen fixation (Sinclair and Krämer, 2012). Membrane recycling of *IRT1* is controlled by ubiquitination in strategy I plants (Barberon et al., 2011). In legumes, Fe uptake and transportation to roots are mainly carried out by protein *HA2*, *FRO2*, and *IRT1* (Walker and Connolly, 2008; Santi and Schmidt, 2009). Putative homologs for the transport of Fe from the leaf to the root through nutrient transporting genes such as *FIT1*, *IRT1*, *OPT3*, and *bZIP23* have been identified in many legumes including peanut (*AhIRT1*; Xiong et al., 2012), *Medicago truncatula* (*MtNRAMP1*; Tejada-Jiménez et al., 2015), soybean (*NRAMP* genes; Qin et al., 2017), lentil (*Ferritin-1*, *BHLH-1*, and FER-like transcription factor protein and *IRT1*), and chickpea (*CaFer1*; Parveen et al., 2016).

Relatively very little is known about Fe uptake, and regulation in legumes shoots (Thomine and Vert, 2013). Fe uptake in shoots is mediated by *IRT*-like transporters, and its movement in the xylem as ferric-citrate complexes has been observed in soybean (Palmer and Guerinot, 2009). Xylem unloading is a crucial step in the distribution and transportation of Fe to different tissues and sinks cell (Figure 2). Expression patterns show that *ZIP* transporters and *YSL* transporters are involved in metal unloading from xylem (Küpper and Kochian, 2010). Oligopeptide transporter (*OPT*) has been suggested to play a significant role in accurate long-distance Fe signaling from shoots to roots and in importing Fe into phloem companion cells in *Arabidopsis* (Kumar et al., 2017). Due to the abundance of nicotianamine (NA) in shoot tissues and its affinity to various ions, it can be assumed that *YSL* transporters are essential for metal transfer from the xylem to the leaves and the seeds, as evident from the expression of *Arabidopsis* genes *AtYSL1* and *AtYSL3* that increased during leaf senescence (Waters et al., 2006). *NRAMP* family genes are known to play a significant role in Fe homeostasis whereas *YSL* and *OPTs* play a major role in loading and unloading of Fe^2+^ NA complexes into and out of phloem (Palmer and Guerinot, 2009). Fe uptake and transportation in plants have been reviewed in several articles (Kobayashi and Nishizawa, 2012; Curie and Mari, 2017).

### Zinc (Zn) Transport

Efficient uptake, transport, and accumulation of Zn in seeds are equally crucial for developing nutrient-rich crops (Astudillo et al., 2013). In legumes, Zn is mostly taken up across the plasma membrane of root cells as Zn^2+^. *ZIP* transporters have been involved in Zn uptake and transport from root to seeds (Colangelo and Guerinot, 2006; Palmgren et al., 2008). *ZRT*, *IRT*-like protein (*ZIP*), HMA heavy metal ATPase (*HMA*), Zinc-induced facilitator (*ZIF*), and metal tolerance protein (*MTP*) have been involved in Zn transport (Hussain et al., 2004). *MTP*s play a role in the mobilization of many metal ions such as Zn, Mn, Fe, Ni, Cd, and Co in the cytoplasm. In the case of *M. truncatula*, *MtZIP1*, *MtZIP3*, *MtZIP4*, *MtZIP5*, *MtZIP6*, and *MtZIP7* genes were found to be upregulated under Zn deficiency in both roots and leaves, suggesting their active role in Zn transport (Hussain et al., 2004). The *bZIP* family is another important gene family involved in Zn transport in legumes. Studies in many dicots such as *Arabidopsis*, soybean (*GmZIP1*), common bean (*PvZIP12*, *PvZIP13*, *PvZIP16*, and *PvbZIP1*), *Medicago* (seven ZIP transporters), and *Lotus japonicus* have identified *ZIP* genes in different tissues like roots, leaves, and seeds (Lin et al., 2009; Astudillo et al., 2013). Mostly, Zn is transported through the symplastic pathway, but a considerable fraction may follow the apoplastic pathway through roots to reach the xylem (White et al., 2002; Figure 2). The cation diffusion facilitator (CDF) family members such as *MTP1* and *ZIF1* transporter play a role in Zn transport to the vacuole while *NRAMPs* have been identified in Zn mobilization from the vacuole (Haydon and Cobbett, 2007). Zn loading to the xylem is mediated through *HMA*, while within the xylem, is transverse as Zn^2+^ or in complex with histidines or Nicotianamine (Palmgren et al., 2008). While *ZIP* family members are actively involved in mediating Zn^2+^ influx to leaf tissue and also to the phloem, *YSL* is involved in loading Zn to the phloem and unloading to the seeds as Zn-NA complex (Haydon and Cobbett, 2007; Waters and Grusak, 2008).

### Manganese (Mn) Transport

Manganese is an essential trace element in plants as it serves as a cofactor in many vital processes such as photosynthesis and lipid biosynthesis. Mn is available in the soil as Mn^2+^ for plant uptake (Figure 2). Very few transporters have been identified exclusively for Mn transport in plants. However, there are many transporters such as *NRAMP*, *YSL*, *IRT1*, *CDF/MTP*, P-Type-ATPase and *VIT* (vacuolar iron transporter) (Xia et al., 2010; Socha and Guerinot, 2014) that help in Mn transport. Transporters in Mn have broad specificity for other divalent cations such as Cd, Ca, Co, Zn, Fe, Cu, and Ni. In *Arabidopsis*, *AtNRAMP1* was reported to be a high-affinity transporter for Mn transport in roots, and knockout lines for *AtNRAMP1* showed susceptibility toward Mn deficiency (Cailliatte et al., 2010). *ZIP1* remobilizes Mn from vacuoles to allow Mn translocation to the shoot through root vasculature (Milner et al., 2013). However, *ZIP2* transporters do not seem to be the primary transporters of Mn in roots of many species, including *M. truncatula*. In the case of field pea and *M. truncatula*, *PsIRT1*, *MtZIP4*, and *MtZIP7* genes can reestablish growth to the Mn uptake defective *smf1* mutant in Mn-limited media indicating *IRT/ZIP* as a direct transporter of Mn in strategy I plants (Milner et al., 2013). A subset of cation channels such as Ca^2+^-permeable channels transport Mn^2+^ in the apical plasma membrane of *Arabidopsis* root hairs (Véry and Davies, 2000; Socha and Guerinot, 2014). Involvement of other routes in Mn transport can be plausible because of the presence of many transporters associated with Mn transport even in the absence of vacuolar iron transporter 1 (*VIT1*).

### Phosphorus (P) Transport

Phosphorus uptake of plants from the soil is in the form of phosphate (P_i_) either via root epidermal cells impelled through a proton gradient produced by plasma membrane H^+^-ATPases or with the help of arbuscular mycorrhizal fungi (AMF) found in legumes (Bucher, 2007; Figure 2). Several *Pht1* genes are expressed in roots, aerial parts, and seeds, implying their potential involvement in internal P_i_ translocation. In the case of *M. truncatula*, P_i_-transporters genes (*MtPT1* and *MtPT2*) from the *Pht1* family were found to be highly expressed in P_i_-deprived roots (Liu et al., 2008). However, only *MtPT5* showed high affinity for P_i_ uptake among the reported five (*MtPT1*, *MtPT2*, *MtPT3*, *MtPT4*, and *MtPT5*) *Pht1* family genes in *M. truncatula* (Liu et al., 2008). In *L. japonicus*, three P_i_ transporter genes of the *Pht1* family have been isolated (Maeda et al., 2006). In the case of soybean, 14 *Pht1* genes (*GmPT1-GmPT14*) were identified in response to P_i_ availability in various tissues associated with its uptake and translocation (Qin et al., 2012). A high-affinity P_i_ transporter, *GmPT5* helps in maintaining P_i_ homeostasis by regulating movement from roots to the region of aerial plant tissues in nodules of soybean (Qin et al., 2012). In chickpea, *CaPHO1*, *CaPHO2*, *CaPHT1;4*, *CaPAP17*, *CaPPase4*, and *CaDGD1* were involved in P_i_ uptake, transport, allocation, and the mobilization/remobilization from roots and leaves to nodules (Esfahani et al., 2016). *Pht1* transporters are mostly involved in transferring P_i_ into cells while other members of the *Pht2*, *Pht3*, and *Pht4* families are associated with the transfer of P_i_ in the intercellular membrane.

### Copper (Cu) Transport

Copper uptake from the soil follows similar strategies like Fe, entering the root cell through copper transporters (COPT) family transporter (Gayomba et al., 2013; Ryan et al., 2013). Cu is mostly available in the soil as Cu^2+^, which is transported to the root cell in its reduced form “Cu^+^” (Figure 2). Ferric reductase, *FRO2*, helps in reduction activity and also in Cu^+^ uptake by roots (Bernal et al., 2012). In *Arabidopsis*, Cu stress induces high Cu^2+^ chelate reductase activity regulated by SPL7, and this reductase was encoded by *FRO4/5* at the root tips (Bernal et al., 2012; Ryan et al., 2013). After reduction, Cu^+^ is transported through the roots by copper transporter (*COPT*) proteins. *COPT* proteins have not been studied in detail in legumes. However, in *Arabidopsis*, *COPT1* (in roots) and *COPT2* (in shoots) are the core uptake transporters whereas *COPT3* and *COPT5* might be involved in intracellular Cu mobilization (Gayomba et al., 2013). Besides, *COPT* transporters *ZIP2* and *ZIP4* are also believed to support Cu uptake in plant cells in *Arabidopsis*. In *Arabidopsis*, the cysteine-rich metallothionein proteins (MT proteins) were upregulated during Cu stress, whereas in field pea, MT mRNA levels were mildly upregulated in Cu stress conditions.

## Metabolic Pathways for Vitamins (β-Carotene, Folate, and Vitamin E) in Legumes

Understanding the pathways to and rate-limiting steps in the accumulation of various seed nutrients is a major challenge. Initial efforts in developing nutrient-rich crops have focused on overexpression of single genes that affect nutrient biosynthesis/uptake, transport or storage. Various studies have suggested that overexpression of a single gene is not sufficient to increase the accumulation of nutrients in seeds (Ishimaru et al., 2010). Considering the complex nature of nutrient accumulation in plants, multiple genes at different steps of translocation or biosynthetic pathways need to be manipulated simultaneously to increase seed nutrient concentrations. To enhance vitamins’ content in legumes, a cohesive understanding of the genetics of nutritional traits along with a knowledge of regulatory biochemical and molecular processes in the accumulation of nutrients are required (Asensi-Fabado and Munné-Bosch, 2010; Bhullar and Gruissem, 2013). A brief description of vitamins such as β-carotene, folate, tocopherol and anti-nutritional components such as phytic acid and raffinose biosynthesis are discussed below.

### Beta (β)-Carotene Biosynthesis

Plant carotenoids are the generic name for C_40_ tetraterpenoids with a conserved biosynthetic pathway that play a significant role in different processes including photosynthesis (DellaPenna and Pogson, 2006). There are two major groups of carotenoids; the first is oxygenated or xanthophyll that consists of lutein, violaxanthin, and neoxanthin, and the second is non-oxygenated or carotenes that include β-carotene and lycopene (DellaPenna and Pogson, 2006). Seeds of legumes are rich in carotenoids such as β-carotene, cryptoxanthin, lutein, and zeaxanthin (Abbo et al., 2005). For instance, β-carotene concentration in chickpea was higher than in genetically engineered “golden rice” endosperm but lower than in Golden Rice2, where β-carotene concentration was increased up to 23-fold (Abbo et al., 2005).

In legumes, plastid-confined MEP (2-C-methyl-D-erythritol 4-phosphate) pathway produces carbon flux, which is used for carotenoid biosynthesis (Giuliano, 2014). Carotenoid concentration is a highly heritable trait which is least affected by the environment (Owens et al., 2014). Identifying the metabolic bottlenecks associated with the carotenoid pathway can help in modifying strategies to develop carotenoid-rich crops. The key regulator gene of the carotenoid pathway is *PSY*; the overexpression of this gene or phytoene desaturase gene individually or a in combination has been practiced in several crops including soybean (Schmidt et al., 2015). In soybean, a 1500-fold increase in β-carotene content in dry seeds was observed compared to wild-type by introducing a chimeric gene from pea and a *crtB* gene from bacterium *Pantoea* using a biolistic method (Schmidt et al., 2015). In chickpea, four members of the *PSY* family that might have a positive effect on carotenoid concentration for various cotyledon colors were reported. A total of 32 genes for isoprenoid and carotenoid pathways in chickpea distributed across all eight chromosomes were also identified (Rezaei et al., 2016). Phytoene synthase and desaturase were found to have a major impact on pro-vitamin A and total carotenoid concentration through genetic transformation or overexpression of these genes. Xanthophylls are produced by converting pro-vitamin A compound with the help of β-carotene hydroxylation and can help in developing cultivars with higher pro-vitamin A as seen in potato, where silencing of β-carotene hydroxylase increased β-carotene concentration (da Silva Messias et al., 2014). Lutein, one of the main carotenoid types in chickpea, showed higher concentration in desi compared to kabuli type and was found to be adversely associated with seed weight (Abbo et al., 2005; Ashokkumar et al., 2014). Carotenoid concentration was higher in genotypes with green cotyledons in both pea and chickpea; a similar trend for lutein was observed in pea. Similarly, in transgenic soybean, increased concentration of β-carotene and seed protein content, with a decreased level of abscisic acid in cotyledons by overexpressing a seed-specific bacterial phytoene synthase gene was observed (Schmidt et al., 2015).

### Folate Biosynthesis

Folates (Tetrahydrofolate and derivatives) are water-soluble B vitamins that act as cofactors in many vital metabolic functions, including the metabolism of amino acids, biosynthesis of nucleic acids in the human body. Legumes are a rich source of folates. A high concentration has been estimated in chickpea (351–589 μg/100 g), common bean (165–232 μg/100 g), and lentil (136–182 μg/100 g), (Blancquaert et al., 2014; Jha et al., 2015). Plants are the only source of folate for humans as the human body cannot synthesize it. Folate biosynthesis takes place in three subcellular compartments. Firstly, the Pterin and pABA moieties are synthesized in cytosol and plastids, respectively, while the rest of the reactions take place in the mitochondria. Pterin moiety synthesizes by converting GTP into dihydroneopterin triphosphate and formate with the help of GTP cyclohydrolase-I (Hossain et al., 2004). In legumes, pABA is synthesized from chorismate through two reactions in plastids. In mitochondria, after pyrophosphorylation of 6-hydroxymethyldihydropterin (HMDHP), it combines with pABA to form dihydropteroate with the help of enzymes HMDHP pyrophosphokinase and dihydropteroate synthase. After this reaction, glutamate residue is combined with the carboxy part of the pABA moiety of dihydropteroate to produce dihydrofolate with the help of enzyme dihydrofolate synthetase. Finally, folate is formed by the attachment of a glutamate tail to THF molecule catalyzed by dihydrofolate reductase.

Considering the complex nature of folate biosynthesis, metabolic engineering has emerged as a better approach to increase folate concentration in plants, such as by the overexpression of genes involved in pterin biosynthesis, a folate biosynthesis precursor (Hossain et al., 2004; Storozhenko et al., 2007; Blancquaert et al., 2014). Around a 150-fold increase in biosynthetic pteridines was reported in transformed lines of the common bean by introducing GTP cyclohydrolase I from *Arabidopsis* in three cultivars by particle bombardment (Rivera et al., 2016).

### Vitamin E Biosynthesis

Tocopherol and tocotrienol derivatives are collectively called vitamin E. Improvement for vitamin E mostly focuses on enhancing vitamin E content in edible parts by regulating the activity of various enzymes involved in different steps of the synthesis, such as p-hydroxyphenylpyruvate dioxygenase, homogentisate phytyltransferase, homogentisate geranylgeranyl transferase, homogentisate solanesyltransferase2-methyl-6-phytyl-benzoquinol methyltransferase, tocopherol cyclase, and γ-tocopherol methyltransferase (Tang et al., 2016). Overexpression of γ-*TMT* resulted in an increased proportion of α-tocopherol in soybean (Sattler et al., 2004; Tavva et al., 2007) while overexpression of both MT and γ-*TMT* increased α-tocopherol 5-folds in soybean (Tavva et al., 2007). Overexpression for the combination of tyrA, HPPD, GGPP reductase and HPT resulted in an 11-fold increase in vitamin E content in soybean (Karunanandaa et al., 2005).

### Metabolic Pathways of Anti-nutrients (Phytic Acid and Raffinose)

Phytic acid binds to mineral cations to form a mixed salt called phytate and sequesters inorganic phosphate in legumes. Myo-inositol is the precursor for many metabolites, including phytate, which plays an important role in plant stress adaptation. In addition to stress response, phytate plays a major role during seed germination to develop embryos and defense against oxidative stress. Considering its anti-nutritional role, breeding and transgenic approaches were used to reduce phytic acid in legumes (see Panzeri et al., 2011; Joshi-Saha and Reddy, 2015). In common bean, genes *PvMIPSs* and *PvMIPSv* (coding for myo-inositol 1phosphate), *PvIMP* (inositol monophosphatase), *PvMIK* (myo-inositol kinase), *PvIPK2* (inositol 1,4,5-tris-phosphate kinase), *PvITPKa* and *PvITPKb* (inositol 1,3,4-triphosphate 5/6-kinase), and *PvIPK1* (inositol 1,3,4,5,6 pentakisphosphate 2-kinase) have been identified and mapped on a reference genetic map through virtual mapping strategy (Fileppi et al., 2010). In common bean, a low phytic acid line (*lpa*1) 280-10 was selected and used for the identification of *Mrp1* gene that down-regulates the phytic acid pathway at the transcriptional level (Panzeri et al., 2011). *lpa* mutants have also been identified in other legumes such as field pea and soybean using EMS-based mutagenesis (Warkentin et al., 2012). In chickpea, *CaMIPS2* gene was found to be regulating the phytic acid biosynthesis pathway (Kaur et al., 2008). In soybean, identification of consistent metabolic changes in *lpa* mutants showed decreased content of myo-inositol and raffinose compared to the wild type and reported a significant role in reducing phytic acid (Frank et al., 2009). Silencing expression of multidrug resistance-associated protein (MRP) ATP-binding cassette (ABC) transporters in an embryo-specific manner resulted in low phytic acid and high inorganic phosphate in transgenic maize and soybean (using homologous soybean MRP gene) (Shi et al., 2007).

Raffinose is another major anti-nutrient affecting plant nutrition potential. In chickpea, raffinose content varied from 0.38 g/100 g to 0.99 g/100 g, while stachyose content ranged from 0.79 g/100 g to 1.87 g/100 g. Synthesis of galactinol is a key requirement for entering into the pathway of the raffinose family of oligosaccharides (RFO) biosynthesis. The key enzyme galactinol synthase synthesizes galactinol using UDP Galactose. Raffinose synthase helps to synthesize raffinose, and stachyose synthase helps to produce tetrasaccharide stachyose by utilizing galactinol, and both these reactions are reversible.

Understanding interactions between micronutrients, such as the synergic effect of Fe and pro-vitamin A carotenoids or the competitive effect of Fe and Zn and bioconversion factors, are essential for the development of nutrient-rich crops. Bioavailability of nutrients depends on endogenous (phytic acid, fiber, amino acids, and proteins) and exogenous factors in seeds. Legumes contain some promoters that enhance the bioavailability of minerals, even in the presence of anti-nutrients. Some promoter compounds are natural plant metabolites, and only minor changes in its accumulation in seeds may be necessary to impact the bioavailability of micronutrients. Inulin is a fructooligosaccharide found in small amounts in raw samples of lentil, chickpea, red kidney bean, common white bean, white bean and faba bean (Rastall and Gibson, 2015). It has a significant positive effect on improving the bioavailability of mineral nutrients in legumes.

Further studies are required to understand the types and amounts of prebiotics concerning in relation to increased bioavailability of minerals. Nicotianamine levels in plants have also shown a positive effect on enhancing Fe concentrations in seeds. Breeders should focus on enhancing the level of promoters such as inulin, β-carotene, histidine, lysine, riboflavin, and selenium, which can increase the bioavailability of Ca, Fe, Zn, Mg, and I (White and Broadley, 2005).

## Agricultural Interventions Through Biofortification

**FIGURE 3:**
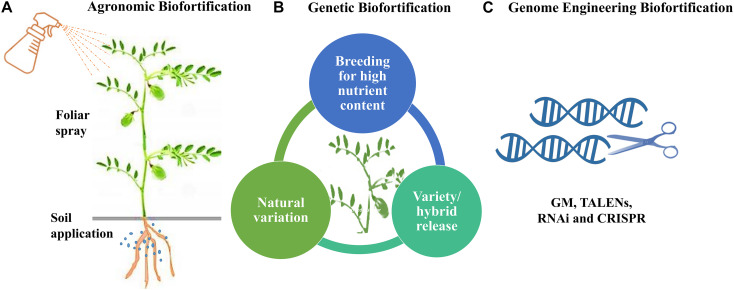
The three approaches for biofortification. **(A)** Agronomic Biofortification using soil and foliar spray. **(B)** Genetic Biofortification through breeding using conventional and genomics-assisted breeding. **(C)** Genome Engineering Biofortification including GM and DNA alteration technologies such as TALENs, RNAi and CRISPR.

Biofortification is the most sustainable approach to increase nutrient concentration and bioavailability in staple food crops. It refers to the procedure of improving the concentration of essential minerals, vitamins, essential amino acids, and fatty acids and reduces anti-nutritional factors enabling nutrient bioavailability in crop plants (Garcia-Casal et al., 2017). Biofortification approaches include the application of fertilizer to the soil or leaves, plant breeding, and genetic engineering (genetic modification and transgenesis) (Figure 3). It is the most economical and cost-effective way to provide nutrient-rich food to most vulnerable people and gives better yield and profit to farmers (Garcia-Casal et al., 2017).

### Agronomic Biofortification

Fertilizer application for macronutrients (N, P, K, and S) and micronutrients (Zn, Ni, I, Co, Mo, and Se) have a significant impact on the accumulation of nutrients in seeds compared to other micronutrient fertilizers such as Fe that have limited phloem sap mobility. The concentration of minerals in the seed and cotyledon can be increased by optimizing the rate and timing of foliar application and using an approach that combines the application of soil and foliar spray to achieve a higher concentration of grain minerals. During foliar application, the minerals get absorbed by the leaf epidermis and then transported to sink via the xylem and phloem. Increasing the available soil concentration of Zn, Ni, I, and Se can significantly increase their concentrations in seeds, as confirmed by a study on pea and navy beans. Accumulation of Zn in the seed of field pea was positively influenced by the combined foliar application of Se and Zn. Se and I concentrations were improved in edible parts through the combined foliar application with increased Se and I (Poblaciones and Rengel, 2017). However, in the case of Fe, fertilization could not affect the mineral content of the seed. Application of fertilizer either in the soil or through foliar spray is a temporary solution compared to breeding approaches. Therefore, fertilizer application can be one of the effective ways to improve the concentration of nutrients in edible parts in combination with breeding or transgenic approaches.

### Genetic Biofortification Through Breeding

Genetic biofortification includes the application of plant breeding techniques to produce crops with higher micronutrient content, moderate to low levels of anti-nutrients, and increased levels of substances that promote nutrient absorption (Bouis, 2003). Breeding approaches have great potential to increase micronutrient density by exploring the existing genetic variation to develop nutrient-rich crop varieties. Considering its sustainability and no regulatory and political restrictions, biofortification through breeding seems to be the most suitable approach for biofortification (Saltzman et al., 2017).

In order to develop a legume genetic biofortification program, the first step involves setting a target micronutrient level for each crop. Among the factors that affect genetic biofortification are available genetic variability and information about genes that control the absorption of the element by roots, translocation to shoots, mobilization in different vegetative parts, and deposition of the element in the edible parts in utilizable forms (Bouis and Welch, 2010). Besides, there are various environmental factors and cultural practices that can affect element accumulation in ds, and dietary factors that affect the absorption and utilization of minerals by the consumer (Bouis and Welch, 2010). Most biofortified products in Asia, Africa, and Latin America have been produced using breeding, while other technologies to develop biofortified products are under development (Garcia-Casal et al., 2017). HarvestPlus^2^ works with several CGIAR and National Agricultural Research centers from Africa, Asia and Latin America to develop and promote high nutrition content biofortified food crops. According to the HarvestPlus Annual Report (2015), several biofortified crops yellow cassava, orange flesh sweet potato with high levels of β-carotene (over 200 mg/g), iron beans (50–70% more iron content), orange maize, iron pearl millet, zinc rice and zinc wheat] developed through breeding have been released officially in more than 30 countries and are in the testing stage in more than 50 countries. Several studies have reported the efficacy of these released biofortified varieties in improving micronutrient deficiency among target populations (De Moura et al., 2014; Finkelstein et al., 2017).

Micronutrients constitute a very small portion of the total weight of a grain legume; therefore, precision estimation is a pre-requisite for effectively assessing genetic variation for breeding with stable and high element concentration. However, quick, accurate, and inexpensive methods for identifying nutrient-dense genotypes are yet to be identified. If wild relatives are found to be the source of micronutrients, pre-breeding approaches can be used to develop the parent for genetic biofortification. Transgressive segregation of heterosis can be exploited to create a genetic variation for the target nutrient trait in case it is difficult to achieve this through selection (Bouis and Welch, 2010). Any breeding program requires an understanding of the genetics of the target trait in order to select the parental line and breeding method. In addition, clarity on the correlation between nutritional traits and yield and yield-related traits will aid the selection of nutrient-rich lines with higher yields and desired traits (Bouis and Welch, 2010). For instance, a positive correlation between Fe and Zn content in seeds of common bean, peanut, mung bean, wheat, pearl millet, maize, and sesame indicates the scope to simultaneous improve these two traits (Cichy et al., 2009; Pixley et al., 2011; Velu et al., 2012). In the case of chickpea, negative correlation between Zn and grain yield across locations was reported, while a significant negative correlation of Fe with grain yield was observed at one location (Diapari et al., 2014).

### Genome Engineering Biofortification

Transgenic approaches are necessary and even advantageous in comparison to breeding in the absence of natural variation in the gene pool of the target crop (Al-Babili and Beyer, 2005). The best example of genome engineering for the nutritional trait is “Golden Rice,” where an advanced transgenic line having 37 mg/g carotenoid was developed (Al-Babili and Beyer, 2005). Despite the positive side of transgenics, researchers have raised concerns related to allergies or intolerance associated with bioengineered or genetically modified crops along with the environmental side effects, and reduced biodiversity (Maghari and Ardekani, 2011; Raman, 2017).

Recent advances in molecular biology have significantly changed the mutagenesis platforms for more targeted and accurate DNA alterations through transcription activator-like effector nucleases (TALENs), zinc-finger nucleases (ZFNs), and short palindromic repeat (CRISPR)–associated protein (Cas9) in legumes and other crops (Curtin et al., 2011; Haun et al., 2014; Michno et al., 2015; Sun et al., 2015; Tang et al., 2016). New technologies like TALENs, ZFNs, RNA interference (RNAi), and CRISPR/Cas9 need to be utilized for the improvement of nutritional traits in legumes. Of the different genetic engineering technologies, RNAi seems to have the edge over others as it is an advanced specific gene silencing technology and a very powerful innovation that can help to develop nutritionally rich and anti-nutrient low crops (Tang and Galili, 2004). RNAi technology has already been used to reduce the level of BOAA in grass pea, to reduce the content of Arah2, an allergen, by 25% in crude peanut extract (Dodo et al., 2008), and develop peanut oils having novel combinations of oleic acid content. RNAi has also been used to generate resistant common bean lines to *Beans Golden Mosaic Virus* (Bonfim et al., 2007). Suppression of *SACPD* gene through RNAi has increased resistance to several pathogens in soybean (Jiang et al., 2009). RNAi interventions targeting genes associated with lignin production resulted in enhanced resistance of soybean to *Sclerotinia sclerotiorum* because of reduced lignin concentration (Peltier et al., 2009). The RNAi approach has also helped in improving oleic acid in soybean. Apart from legumes, it has also been used to improve nutritional quality in maize, wheat, rice, cotton, jute, and tomato (Kusaba et al., 2003; Davuluri et al., 2005). Table 3 summarizes the list of genetic modifications that have taken place so far in legume biofortification.

**TABLE 3:** Studies on genetic modification in legumes for biofortification.

Crop	Nutritional trait	References
Soybean	Amino acid	Falco et al., 1995; Reddy and Thomas, 1996; Kita et al., 2010
	Fats and oils	Kinney and Clemente, 2005; Clemente and Cahoon, 2009
	Low Phytase	Denbow et al., 1998; Chiera et al., 2004; Bilyeu et al., 2008
	Vitamin E	Van Eenennaam et al., 2003; Sattler et al., 2004
	Flavonoids	Yu et al., 2003
	Low Phytate	Yuan et al., 2007
	Plenish high oleic	Plenish high oleic, 2021
	Vistive Gold low saturated high oleic	Monsanto; Ulmasov et al., 2012
Common bean	Lysine	Falco et al., 1995
	Folate	Rivera et al., 2016; Xie et al., 2017
	Low Phytate	Panzeri et al., 2011
Lupin	Methionine	White et al., 2001

## Genomics Approaches to Nutritional Breeding

Genetic biofortification efforts through breeding methods have been partially effective in addressing the challenge of low nutrient content, though not to the extent desired. Therefore, it is essential to exploit the potential of genomics to accelerate the development of nutrition-rich improved cultivars. Details about genetic and genomic resources for important legumes have been extensively reviewed (Pandey et al., 2016; Bevan et al., 2017; Varshney et al., 2018; Roorkiwal et al., 2020). In the recent past, advancements in next-generation sequencing (NGS) technologies have led to a drastic reduction in cost and thereby resulted in making available genomic sequence for major legumes, enabling NGS-based methods for allele mining, candidate genes identification, and high-resolution genetic mapping. Though cost-effective genotyping platforms are available for deploying genomics-assisted breeding (GAB) in major legumes, the cost of high throughput and efficient estimation of nutrients poses a major challenge. The plant genome sequence offers an opportunity to dissect and understand the mechanism for functional characterization of genes involved in nutrient uptake and mobilization. Among legumes, the genome sequence of pigeon pea (Varshney et al., 2012), chickpea (Varshney et al., 2013), peanut (Bertioli et al., 2016; Chen et al., 2016), lupin (Hane et al., 2017), soybean (Schmutz et al., 2010), and common bean (Schmutz et al., 2014) have been completed and can provide the foundation for deploying genomics in legume breeding by detecting the genes responsible for nutritional traits.

### Exploiting Genetic Variation of Micronutrients in Legumes

Screening of diverse germplasm is a pre-requisite to understanding the genetic variation for a trait of interest that can be used for breeding to increase the availability of that particular element (McCouch et al., 2013). Genetic variation enables a breeder to exploit heterosis, additive gene effects, and transgressive segregation to improve micronutrient concentration. When the required genetic variation is not available, transgenic approaches can provide additional sources of variation (Francis et al., 2017). Legumes are considered a rich source of nutrients and possess huge variation in the legume germplasm (Table 4). Generally, inductively coupled plasma-Mass spectrometry (ICP-MS) is utilized for mineral estimation; however, it requires expensive equipment, a skilled analyst, and extensive sample preparation. The colorimetric approach that has been used to measure minerals is semi-quantitative and laborious when applied for large-scale screening. In atomic absorption spectrometry (AAS), free atoms absorb light in the form of optical radiation for the quantitative detection of elements present in a sample. X-ray fluorescence spectroscopy (XRF) is also a consistent, high throughput, low-cost system to determine element concentrations in samples; it is classified as being either energy dispersive (EDXRF) or wavelength dispersive (WDXRF) (Singh et al., 2013).

**TABLE 4:** Macronutrient content in some legumes (per 100 g).

	Macronutrient	Common bean	Chickpea	Cowpea	Pigeon pea	Faba bean	Mung bean	Soybean	Peanut	Lentil	Navy bean
	**Water**	G	13.4	7.7	11.1	10.6	11.0	9.1	8.56.5	8.3	12.1
	**Energy**	kcal	329.0	378.0	343.0	343.0	341.0	347.0	446.0567.0	352.0	337.0
	**Protein**	G	19.9	20.5	23.9	21.7	26.1	23.9	36.525.8	24.6	22.3
	**Total lipid (fat)**	g	0.5	6.0	2.1	1.5	1.5	1.2	19.949.2	1.1	1.5
	**Ash**	g	3.3	2.9	3.4	3.5	3.1	3.3	4.92.3	2.7	3.3
	**Carbohydrate, by difference**	g	62.9	63.0	59.6	62.8	58.3	62.6	30.216.1	63.4	60.8
	**Fiber, total dietary**	g	12.7	12.2	10.7	15.0	25.0	16.3	9.38.5	10.7	15.3
	**Total sugar**	g	–	10.7	–	–	5.7	6.6	7.34.7	2.0	3.9
**Minerals**	**Calcium (Ca)**	mg	66.0	57.0	85.0	130.0	103.0	132.0	277.092.0	35.0	147.0
	**Iron (Fe)**	mg	5.0	4.3	10.0	5.2	6.7	6.7	15.74.6	6.5	5.5
	**Magnesium (Mg)**	mg	127.0	79.0	333.0	183.0	192.0	189.0	280.0168.0	47.0	175.0
	**Phosphorus (P)**	mg	381.0	252.0	438.0	367.0	421.0	367.0	704.0376.0	281.0	407.0
	**Potassium (K)**	mg	1254.0	718.0	1375.0	1392.0	1062.0	1246.0	1797.0705.0	677.0	1185.0
	**Sodium (Na)**	mg	5.0	24.0	58.0	17.0	13.0	15.0	2.018.0	6.0	5.0
	**Zinc (Zn)**	mg	5.0	2.8	6.1	2.8	3.1	2.7	4.93.3	3.3	3.7
	**Copper (Cu)**	mg	1.1	0.7	1.1	1.1	0.8	0.9	1.71.1	0.8	0.8
	**Manganese (Mn)**	mg	1.7	21.3	1.5	1.8	1.6	1.0	2.51.9	1.4	1.4
	**Selenium (Se)**	μg	3.1	0.0	9.1	8.2	8.2	8.2	17.87.2	0.1	11.0
**Amino Acid**	**Tryptophan**	g	0.2	0.2	0.3	0.2	0.2	0.3	0.60.3	0.2	0.2
	**Threonine**	g	0.7	0.8	0.9	0.8	0.9	0.8	1.80.9	0.9	0.7
	**Isoleucine**	g	0.8	0.9	1.0	0.8	1.1	1.0	2.00.9	1.1	1.0
	**Leucine**	g	1.7	1.5	1.8	1.5	2.0	1.8	3.31.7	1.8	1.7
	**Lysine**	g	1.5	1.4	1.6	1.5	1.7	1.7	2.70.9	1.7	1.3
	**Methionine**	g	0.2	0.3	0.3	0.2	0.2	0.3	0.50.3	0.2	0.3
	**Cystine**	g	0.2	0.3	0.3	0.3	0.3	0.2	0.70.3	0.3	0.2
	**Phenyl alanine**	g	1.1	1.1	1.4	1.9	1.1	1.4	2.11.4	1.2	1.2
	**Tyrosine**	g	0.6	0.5	0.8	0.5	0.8	0.7	1.51.0	0.7	0.5
	**Valine**	g	1.0	0.9	1.1	0.9	1.2	1.2	2.01.1	1.2	1.2
	**Arginine**	g	1.3	1.9	1.7	1.3	2.4	1.7	3.23.1	1.9	1.0
	**Histidine**	g	0.5	0.6	0.7	0.8	0.7	0.7	1.10.7	0.7	0.5
	**Alanine**	g	1.2	0.9	1.1	1.0	1.1	1.1	1.91.0	1.0	0.9
	**Aspartic acid**	g	2.4	2.4	2.9	2.1	2.9	2.8	5.13.1	2.7	2.6
	**Glutamic acid**	g	3.1	3.6	4.5	5.0	4.4	4.3	7.95.4	3.8	3.1
	**Glycine**	g	0.8	0.9	1.0	0.8	1.1	1.0	1.91.6	1.0	0.8
	**Proline**	g	0.9	0.8	1.1	1.0	1.1	1.1	2.41.1	1.0	1.1
	**Serine**	g	1.0	1.0	1.2	1.0	1.2	1.2	2.41.3	1.1	1.2
**Vitamins**	**Vitamin C**	mg	0.0	4.0	1.5	0.0	1.4	4.8	6.00.0	4.5	–
	**Thiamin**	mg	0.5	0.5	0.7	0.6	0.6	0.6	0.90.6	0.9	0.8
	**Riboflavin**	mg	0.2	0.2	0.2	0.2	0.3	0.2	0.90.1	0.2	0.2
	**Niacin**	mg	2.6	1.5	2.8	3.0	2.8	2.3	1.612.1	2.6	2.2
	**Pantothenic acid**	mg	1.5	1.6	1.5	1.3	1.0	1.9	0.81.8	2.1	0.7
	**Vitamin B6**	mg	0.4	0.5	0.4	0.3	0.4	0.4	0.40.3	0.5	0.4
	**Folate, total**	μg	622.0	557.0	639.0	456.0	423.0	625.0	375.0240.0	479.0	364.0
	**Choline, total**	μg	–	99.3	–	–	95.8	97.9	115.952.5	96.4	87.4
	**Vitamin A, RAE**	μg	1.0	3.0	2.0	1.0	3.0	6.0	1.00.0	2.0	0.0
	**Retinol**	μg	0.0	0.0	0.0	0.0	0.0	0.0	0.00.0	0.0	0.0
	**Vitamin A, IU**	IU	17.0	67.0	33.0	28.0	53.0	114.0	22.00.0	39.0	0.0
	**Betaine**	mg	–	–	–	–	–	–	2.10.6	–	0.1
	**Beta-Carotene**	μg	–	40.0	–	–	32.0	68.0	13.00.0	23.0	0.0
	**Vitamin E (α-tocopherol)**	mg	–	0.8	–	–	0.1	0.5	0.98.3	0.5	0.0
	**Vitamin K (phylloquinone)**	μg	–	9.0	–	–	9.0	9.0	47.00.0	5.0	2.5

*Source: U.S. Department of Agriculture, Agricultural Research Service. FoodData Central, 2019. fdc.nal.usda.gov.*

### Identification of QTLs/Genes to Interpret Genetic Architecture Concerning Nutrient Accumulation

Linking genetic data with data on nutrition content is an advanced and accurate approach to identify quantitative trait loci (QTLs) associated with a trait of interest. Nutritional profiling of genotypically characterized diverse set of germplasm (core collection, mini-core collection, a reference set, composite set) can link genetic data to global mineral nutrition (Ghandilyan et al., 2009; Norton et al., 2010). Recently, QTL mapping has been widely used to associate genetic variation with phenotypic variation and provide a reliable tool for gene discovery. The associated region or linked region (i.e., genetic markers) identified through QTL mapping can then be isolated or cloned for identification and analysis of the genes concerned. Molecular mapping of the genome segments that govern nutrient content/concentration has been done in many legumes (Table 5). Studies on understanding genes and processes to improve seed nutritional composition by identifying QTLs were limited to a few nutrients in legumes. For instance, QTLs for seed element concentration has been identified in *L. japonicus* (Klein and Grusak, 2009), *M. truncatula* (Sankaran et al., 2009), common bean (Blair et al., 2009, 2010, 2011; Cichy et al., 2009; Casañas et al., 2013), soybean (Zhang et al., 2009; Jegadeesan et al., 2010; Ramamurthy et al., 2014), chickpea (Sab et al., 2020), and lentil (Aldemir et al., 2017). Most of the studies conducted so far to map and tag the gene(s)/QTL(s) controlling micronutrient status in legumes were mostly found to have a quantitative mode of inheritance (Blair et al., 2010). For instance, in the case of *Proteus vulgaris*, two genes (*PvIRT1* and *PvIRT2*) on chromosome-3 and two genes (*PvbZIP2* and *PvbZIP3*) on chromosome-11 were aligned with QTLs for Fe and Zn (Jiang et al., 2008). The list of identified QTLs in several legumes for various nutritional traits has been presented in Table 5.

**TABLE 5:** List of QTLs identified for key nutritional traits in some legumes.

Species	Trait(s)	Published QTL Symbol	Cross	Population	References
Chickpea	Seed Fe and Zn	11 QTLs for seed Fe and 8 QTLs for Zn	MNK-1 × Annigeri 1	F_2:3_	Sab et al., 2020
	Carotenoid (β-carotene, β-cryptoxanthin, β-carotene, lutein)	5 QTLs	CDC Jade × CDC Frontier; CDC Cory × CDC Jade; ICC 4475 × CDC Jade	F_2_	Rezaei et al., 2019
	Seed Fe and Zn concentration	CaqFe1.1; CaqFe3.1; CaqFe4.1; CaqZn2.1; CaqZn3.1; CaqFZ4.1; CaqFZ7.1	ICC 4958 × ICC 8261	RIL	Upadhyaya et al., 2016
	Protein content	4 QTLs	Germplasm	187 genotypes	Jadhav et al., 2015
	Seed protein concentration	3 QTLs	Carneval × MP 1401	RIL	Tar’an et al., 2004
	Protein, Fe, Zn and several macro- and micro-nutrients	119 marker trait associations for 11 nutrition component traits	280 diverse accessions	Germplasm collection	unpublished
*Cicer* sp.	β-carotene content; lutein concentration	BC.QTL1; LC.QTL1	Hadas × Cr 205	F_2_	Abbo et al., 2005
Common bean	Ca, Fe, Zn, and tannins	–	Wide cross	–	Guzmán-Maldonado et al., 2003
	Fe and Zn	26 QTLs	DOR 364 × G 19833	RIL	Blair et al., 2009
	Fe reductase activity; Zn concentration; Fe concentration; net phytate content (mg/seed); net P content (mg seed/g)	Zn-ICPb11.2 (K126G); Fe-ICPb11.1 (BMd33); BM209–BMd4033; E070.9–BM4643	DOR 364 × G 19833	RIL	Blair et al., 2010
	P accumulation	P accumulation	G 19833 × DOR 364	F_5:7_ RIL	Beebe et al., 2006
	Seed coat Ca; seed coat Mg	Ca1 (McatEtc46); Ca7 (P gene); Ca9 (McagEac7); Mg7xc (P gene)	Xana × Cornell 49242	RIL	Casañas et al., 2013
	Seed Fe and Zn concentration	13 QTLs	G14519 × G4825	RIL	Blair et al., 2010
	Seed Fe content; seed Zn content	Fe_cont8.1; Zn_cont5.1	Cerinza × (Cerinza × G 10022)	Advanced Backcross	Blair and Izquierdo, 2012
	Seed mineral	–	G 21242 × G 21078	RIL	Blair et al., 2011
	Seed P concentration; seed phytic acid (%); seed Fe concentration; seed Zn concentration	ATA4; PVctt1; AGAT05; fin	AND 696 × G 19833	RIL	Cichy et al., 2009
	Protein, Zn, Ca and Fe Bioavailability (FeBIO)	QTL for cooked seed protein, Zn, Ca, and FeBIO	206 diverse accessions	Germplasm collection	Katuuramu et al., 2018
	Zn seed content	*Phvul001G233500*	192 diverse genotypes	Germplasm collection	Caproni et al., 2020
	Total condensed tannin concentration	Seed coat tannin 1-1; Seed coat tannin, insoluble 1-1; 1-2; 1-3; 1-1	Andean × Mesoamerican Genepools	RIL	Caldas and Blair, 2009
Garden pea	Protein content	5 QTLs	Wt 10245 × Wt 11238	RIL	Irzykowska and Wolko, 2004
Lentil	Seed Fe and Zn concentration	FeQTL1.1-1.3; FeQTL2.1-2.3; FeQTL4.1-4.6; FeQTL5.1-5.4; FeQTL6.1-6.2; 7.1-7.3	ILL 8006 × CDC Milestone	RIL	Aldemir et al., 2017
*Lotus japonicus*	Seed nutrients	QTLs (55 markers)	MiyakojimaMG-20 × GifuB-129	RIL	Klein and Grusak, 2009
	Ferric reductase activity	1 major QTL	MiyakojimaMG-20 × GifuB-129	RIL	Klein et al., 2012
Medicago	Seed mineral concentrations	46 QTLs	Jemalong-6 × DZA315.16	RIL	Sankaran et al., 2009
Mung bean	P compounds in the seed	2 for phytic acid; 4 for inorganic P; 1 for total P	V1725BG × AusTRCF321925	F_2_	Sompong et al., 2012
Navy Beans	Seed Fe, Zn, P, and phytic acid	Co-localized QTLs for seed Fe and Zn on three linkage groups	AND 696 × G 19833	F_5:7_ RIL	Cichy et al., 2009
	Zn	1 QTL for Fe	Narrow cross Mesoamerican genepool	RIL	Gelin et al., 2007
Field pea	Protein content	prot1	Wt10245 × Wt11238	F_2_	Irzykowska and Wolko, 2004
		PC.LGIII.cccc18.E_2000	Carneval × MP1401	RIL	Tar’an et al., 2004
	Raffinose and for glucose concentration	Two QTLs (RafCleS2.c and GlcT2.b)	champagne × Terese	RIL	Dumont et al., 2009
	Raffinose content; Rubisco content	RafCleS2.c; RuBisCOcleS2	Terese × Champagne	RIL	Dumont et al., 2009
	Seed mineral concentration	37 seed mineral content QTLs	Kiflica × Aragorn	RIL	Ma et al., 2017
Peanut	Linoleic acid; oil content; Oleic acid; O/L ratio; protein content	Seed linoleic 1-1; Seed oleic 1-13; Seed oleic/linoleic 1-1; Seed protein 1-1	TG26 × GPBD4	RIL	Sarvamangala et al., 2011
Soybean	Seed mineral, cysteine, and methionine concentrations	82 × DSR-173; Williams 82 × Vinton 81	Williams82 × NKS19-90	RIL	Ramamurthy et al., 2014
	Seed Ca	Ca1; Ca2; Ca3; Ca4	SS-516 × Camp	F_2:3_	Zhang et al., 2009
	Seed Cd	Major QTL on LG K	AC Home Westag-97	RIL	Jegadeesan et al., 2010
		SSR marker linked with Cd locus	Leo Westag-97	RIL	Jegadeesan et al., 2010
	Vitamin E	4 QTLs for α-Toc; 8 for γ-Toc; 4 for δ-Toc; 5 for Vitamin E	OAC Bayfield × Hefeng 25	RIL	Li et al., 2010
	Minerals in seeds	8 QTLs for K; 4 for Mg; 1 for P; 1 for C; 1 each for N, S, and Ca	MD 965722 × Spencer	RIL	Bellaloui et al., 2017
	Nutritional traits	40 QTLs	Williams 82 × DSR-173, Williams 82 × NKS19-90, and Williams 82 × Vinton 81	RIL	Ramamurthy et al., 2014

In addition to conventional bi-parental mapping populations, efforts have also been made to exploit the available genetic variation for nutrient factors using genome-wide association studies. This approach has been used to identify markers associated with various key nutrition factors in common bean (Katuuramu et al., 2018; Caproni et al., 2020) and chickpea (unpublished). These identified genes/QTLs, after validation, may be deployed in to develop nutrient-rich legumes.

## Prospects of the Role of Genomics in Nutritional Breeding

Next-generation sequencing-based genotyping technologies can be employed to understand the genetics of nutritional traits using precise marker-trait association (MTA), gene discovery, and functional marker development. Their potential has been proven for various agronomic traits in genetic mapping, marker-assisted selection (MAS), and genomic selection (GS) (Varshney et al., 2014, 2019). GAB approaches such as marker-assisted backcrossing (MABC) and marker-assisted recurrent selection (MARS) can be used for the improvement of single or multiple nutritional traits. Considering their higher cost, difficulty in estimation and the complex genetic mechanism controlling nutritional traits, deploying GS could be beneficial.

Next-generation sequencing-based high-density genotyping methods such as genotyping by sequencing (GBS) and whole-genome re-sequencing (WGRS) enable the identification of large-scale genome-wide SNPs for high resolution genetic and association mapping. For instance, in chickpea, kabuli reference genome and *de novo*-based GBS assays were used to identify high-quality SNPs for seed Fe and Zn content from 92 desi and kabuli chickpea accessions (Upadhyaya et al., 2016). Similarly, WGRS data on 300 lines from a chickpea reference set (Varshney et al., 2019) along with nutrient content estimation data is being used to identify markers associated with several key nutrient elements (unpublished data). Furthermore, 3000 lines from the global chickpea composite collection are being studied for micro- and macro-nutrient traits and re-sequenced in parallel to identify novel alleles associated with different nutrients (Varshney, 2016). In addition, recently popularized sequencing-based mapping approaches such as “QTL-Seq,” “MutMap,” “Seq-BSA,” “Indel-Seq,” and “Bulked segregant RNA-Seq (BSR-Seq)” can be adopted for mapping nutritional traits. Unique functional allelic variations selected from candidate genes were found to be linked with seed Fe and Zn concentrations in chickpea (Diapari et al., 2014). In the case of soybean, three candidate genes related to seed Fe and Zn storage in maturing seeds have been identified (Liu et al., 2011). In lentil, two SNP markers closely associated with seed Fe and Zn concentrations have been identified (Khazaei et al., 2017).

In addition to trait mapping, transcriptome sequencing has emerged as an alternative to genome sequencing for targeted expressed gene sequencing. Transcriptome sequencing provides an understanding of gene function and the molecular basis of various components related to nutrient mobilization in crops. Identification of candidate genes associated with nutritional traits is plausible from gene expression profiling data of transcriptome assemblies (Pandey et al., 2016). Expression pattern studies in several legume crops have identified genes involved in nutrient mobilization (Küpper and Kochian, 2010; Conte and Walker, 2011). The emerging and promising areas of proteomics that includes proteome mapping, comparative proteomics, post-translational modification, and protein-protein interaction could assist in future nutritional breeding programs (Pandey et al., 2016; Roorkiwal et al., 2020).

Furthermore, metabolomics-assisted breeding can greatly supplement the present breeding strategy for nutritional traits (Hossain et al., 2004; Storozhenko et al., 2007; Fernie and Schauer, 2009; Blancquaert et al., 2014). A complete study of metabolites is required to dissect the genetic basis of metabolic diversity in legumes. Several studies on plant metabolites have been carried out in crops like *Arabidopsis*, rice, and maize (Keurentjes et al., 2006; Schauer et al., 2006; Chan et al., 2010). The information on the genetic and molecular bases of natural variation in legume metabolomes is still limited. Metabolic profiling for phenylpropanoid and isoflavonoid biosynthesis in *Medicago* has been reported (Farag et al., 2008). The combination of metabolomics with transcriptomics, high-throughput phenotyping, and bioinformatics tools will enable the detection of candidate genes for nutritional traits.

Along with the study of metabolomics, “ionome” profiling is equally important to gain deeper insights into a physiological mechanism related to nutrient accumulation in seeds (Salt et al., 2008). To estimate mineral/micronutrients and their complex networks, ionomics has emerged as a potential area that enables genome-wide understanding of the dynamics of element accumulation in living systems (Baxter, 2010). It helps identify transporters, sensors, and other components that control the expression of metal transport proteins in legumes (Lahner et al., 2003). Ionome also assists in providing information about gene networks regulating various developmental and physiological processes related to the “ionome” of an individual and ultimately leading to the identification of potential candidate genes involved in element uptake, transport, and storage. Identified genes can be incorporated to develop nutrient-rich crops either through genetic modification or molecular breeding. Details about plant ionome have been extensively reviewed (Salt et al., 2008; Baxter, 2010; Huang and Salt, 2016). To sum up, an integrated approach that combines genomics with proteomics and metabolomics has the potential to identify the true candidate that can be directly deployed using GAB to develop nutrient-rich legume varieties.

## Conclusion

The incredible advances in plant nutritional genomics provide effective and long-term solutions to the increasing problem of malnutrition. Efforts should be dedicated to identifying candidate genes using MTA and validation and understanding the genetic mechanism of nutrient uptake in crops. Modern breeding techniques like MAS and GS must be used to develop superior nutritionally rich genotypes. Many other modern technologies such as cisgenesis or intragenesis, RNAi, novel DNA editing technologies such as site-directed mutagenesis, and oligonucleotide-directed changes could be deployed to accelerate the process of varietal development. The focus should be not just on identifying nutrient-rich genotypes but also on the bioavailability of the target nutrient. Therefore, joint research efforts from breeders, biotechnologists, physiologists, and nutritionists are required to support and accelerate biofortification programs in legumes.

## Author Contributions

RKV conceived the idea and provided critical inputs to the concept. MR and SP drafted sections of the manuscript and prepared figures. DT, RH, and RKV made a critical revision of the content of the manuscript. All authors contributed to the final reading and approved the submitted version.

## Conflict of Interest

The authors declare that the research was conducted in the absence of any commercial or financial relationships that could be construed as a potential conflict of interest.

## Footnotes

1 http://www.fao.org/3/I9553EN/i9553en.pdf

2 https://www.harvestplus.org

## Supplementary Material

Supplementary File 1 Recommended Dietary Allowances (RDAs) for various nutrients.
